# Inhibition of apelin expression switches endothelial cells from proliferative to mature state in pathological retinal angiogenesis

**DOI:** 10.1007/s10456-013-9349-6

**Published:** 2013-05-03

**Authors:** Atsushi Kasai, Yuki Ishimaru, Kosuke Higashino, Kohei Kobayashi, Akiko Yamamuro, Yasuhiro Yoshioka, Sadaaki Maeda

**Affiliations:** 1 Interdisciplinary Program for Biomedical Sciences, Institute for Academic Initiatives, Osaka University, 1-6 Yamadaoka, Suita, Osaka, 565-0871 Japan; 2 Laboratory of Molecular Neuropharmacology, Graduate School of Pharmaceutical Sciences, Osaka University, Osaka, 565-0871 Japan; 3 Department of Pharmacotherapeutics, Faculty of Pharmaceutical Sciences, Setsunan University, Osaka, 573-0101 Japan

**Keywords:** Apelin, Angiogenesis, MCP-1, Pericytes, Vascular endothelial cells, Smad

## Abstract

The recruitment of mural cells such as pericytes to patent vessels with an endothelial lumen is a key factor for the maturation of blood vessels and the prevention of hemorrhage in pathological angiogenesis. To date, our understanding of the specific trigger underlying the transition from cell growth to the maturation phase remains incomplete. Since rapid endothelial cell growth causes pericyte loss, we hypothesized that suppression of endothelial growth factors would both promote pericyte recruitment, in addition to inhibiting pathological angiogenesis. Here, we demonstrate that targeted knockdown of apelin in endothelial cells using siRNA induced the expression of monocyte chemoattractant protein-1 (MCP-1) through activation of Smad3, via suppression of the PI3K/Akt pathway. The conditioned medium of endothelial cells treated with apelin siRNA enhanced the migration of vascular smooth muscle cells, through MCP-1 and its receptor pathway. Moreover, in vivo delivery of siRNA targeting apelin, which causes exuberant endothelial cell proliferation and pathological angiogenesis through its receptor APJ, led to increased pericyte coverage and suppressed pathological angiogenesis in an oxygen-induced retinopathy model. These data demonstrate that apelin is not only a potent endothelial growth factor, but also restricts pericyte recruitment, establishing a new connection between endothelial cell proliferation signaling and a trigger of mural recruitment.

**Electronic supplementary material:**

The online version of this article (doi:10.1007/s10456-013-9349-6) contains supplementary material, which is available to authorized users.

## Introduction

While the factors and mechanisms involved in the formation of endothelial tubes [1] and endothelial-pericyte interactions [2] are well characterized, relatively little is known regarding the events that trigger vessel maturation to newly formed endothelial tubes. Tip cells are localized at the leading edge of sprouting vessels and secrete several factors. These are followed by proliferating endothelial stalk cells, which create the capillary lumen [3]. In this process, the further the newly formed patent vessel with an endothelial lumen is from the tip cells, the less proliferative signals it receives. Moreover, pericyte recruitment occurs after the formation of the endothelial lumen [4]. Based on this, we hypothesized that decreased proliferative signals in newly-formed endothelial tube may be an important trigger for the secretion of several factors, inducing pericyte migration.

Apelin is an endogenous ligand for the G protein-coupled receptor APJ, which was recently identified as a marker of mesodermal populations containing angiohematopoietic and mesenchymal stem/stromal cells [5, 6]. During angiogenesis, hematopoietic stem cells and progenitor cells can induce production of apelin from endothelial cells [7]. Recently, much attention has been focused on the possible role of the apelin-APJ system in physiological or pathological angiogenesis [8–11]. This system is involved in the proliferation of endothelial cells, independently of VEGF/VEGFR2 signaling [12, 13]. We previously reported that APJ is selectively expressed in endothelial cells, particularly in the vascular plexus where many endothelial cells are proliferative. In this study we demonstrate that the up-regulation of apelin expression causes exuberant endothelial cell proliferation and pathological angiogenesis in the retinas of mice with oxygen-induced retinopathy (OIR) [13], a frequently used model for ischemic retinal diseases [14]. Therefore, we hypothesize that inhibition of apelin signaling in endothelial cells may induce pericyte recruitment and suppress endothelial cell proliferation, thus inhibiting pathological retinal angiogenesis.

Monocyte chemoattractant protein-1 (MCP-1) is a member of the C–C chemokines, which promote the recruitment and activation of monocytes and macrophages, critically contributing to the inflammatory reaction process in various diseases [15]. MCP-1 is localized in endothelial cells in proliferative diabetic retinopathy [16], and its regulation by high glucose levels in vascular cells has been implicated in the pathogenesis of the inflammatory process associated with diabetes [17]. In addition, MCP-1 directly contributes to tumor angiogenesis via a mechanism independent of monocyte recruitment [18], and also stimulates mural cell recruitment [19, 20]. To date, however, there are no studies focused on the function of this chemokine in mural cell recruitment during pathological retinal angiogenesis.

Here, we show that targeted knockdown of apelin using siRNA in endothelial cells, induced the expression of MCP-1 via phosphorylation and nuclear translocation of Smad3, by suppression of the phosphatidylinositol 3-kinase (PI3K)/Akt pathway. We also demonstrate that induction of MCP-1 by apelin siRNA promoted the migration of vascular smooth muscle cells. Moreover, in vivo delivery of apelin siRNA to the retina resulted in increased pericyte coverage and suppressed pathological angiogenesis in a murine model of OIR. Our data establish a new connection between endothelial cell proliferation and mural recruitment under pathological conditions.

## Methods

### Cell culture

Mouse brain endothelioma bEnd.3 cells were maintained in high glucose (4.5 g/L) Dulbecco’s modified Eagle’s medium (DMEM) containing 10 % fetal bovine serum (FBS). Mouse vascular smooth muscle P53LMAC01 cells were purchased from Health Science Research Resources Bank (Cat. No. JCRB0150, Japan Health Science Foundation) and grown in high glucose (4.5 g/L) DMEM containing 10 % FBS. Cell lines were cultured at 37 °C with 5 % CO_2_. For in vitro RNAi experiments, cells were transfected with 20 μM siRNA using Lipofectamine 2000 (Invitrogen Life Technologies, Carlsbad, CA) according to the manufacturer’s instructions. To inhibit the PI3K/Akt pathway or Smad3 signaling in endothelial cells, LY284002 (3 μM) (PI3K inhibitor; Cell Signaling Technology Inc., Danvers, MA) or SIS3 (3 μM) (Smad3 inhibitor; Santa Cruz Biotechnology Inc, Santa Cruz, CA) was added to culture media at the indicated concentrations.

### Real-time reverse-transcription polymerase chain reaction (RT-PCR)

Total RNA was isolated from cells or retinas using the SV total RNA isolation kit according to the manufacturer’s instructions (Promega Corporation, Madison, WI). Reverse transcription of total RNA (1 μg) was performed as previously described [13]. Quantification of all gene transcripts was conducted using quantitative real-time RT-PCR with an ABI PRISM 7900 HT (Applied Biosystems Life Technologies, Foster City, CA). Real-time RT-PCR was performed using SYBR premix Ex Taq II (Takara, Ohtsu, Japan). Data are expressed as arbitrary units normalized to β-actin (ACTB). The sequences of the PCR primer pairs used for each gene are shown in Supplementary Table S1.

### Western blotting

Cells were lysed in lysis buffer (20 mM Tris–HCl, 2 % sodium dodecyl sulfate, 10 mM NaF, 1 mM Na_3_VO_4_) containing protease inhibitors (1 mM PMSF and 1 μg/mL aprotinin) and centrifuged to remove the insoluble fraction. Lysates were subjected to sodium dodecyl sulfate polyacrylamide gel electrophoresis and electro-transferred to polyvinylidene difluoride membranes for immunoblotting [21]. Membranes were blocked with 1 % bovine serum albumin or 1–5 % nonfat dried milk in Tris-buffered saline (TBS) containing 0.05 % Tween-20 and probed with antibodies recognizing MCP-1 (Rabbit polyclonal, Abcam Inc., Cambridge, MA), Smad3 phospho pS423 pS425 (Rabbit polyclonal, Rockland Inc., Pennsylvania, PA), Smad3 (C67H9) (Rabbit monoclonal, Cell Signaling Technology Inc.), Phospho-Akt (Ser473) (D9E) (Rabbit monoclonal, Cell Signaling Technology Inc.), Akt (Rabbit polyclonal, Cell Signaling Technology Inc.) or beta actin horseradish peroxidase (HRP) (8226) (Mouse monoclonal, Abcam). Primary antibodies were detected with anti-rabbit IgG HRP-linked antibody (Cell Signaling Technology Inc.) and Western Lighting (Perkin-Elmer Inc., Waltham, MA).

### Immunocytochemistry

Cells were fixed with 4 % paraformaldehyde/phosphate-buffered saline (PBS) solution and permeabilized with TBS containing 0.1 % Triton X-100. Immunocytochemistry was performed using anti-Smad3 pS423 pS425 antibody (Rockland Inc.), and Alexa Fluor 568-conjugated goat anti-rabbit IgG (Molecular Probes Life Technologies, Eugene, OR). Nuclear staining was performed using Hoechst 33342 (Sigma-Aldrich, St. Louise, MO).

### Scratch assay

A confluent vascular smooth muscle cell (VSMC) monolayer in a 24-well plate was wounded by manually scraping the cells with a blue pipette tip. VSMCs were treated with conditioned medium (CM) from endothelial cells exposed to control or apelin siRNA for 48 h. Thymidine (5 mM) (Sigma-Aldrich) was included during the incubation period to inhibit VSMC proliferation. To examine the role of MCP-1/CCR2 signaling in VSMC recruitment, the CCR2 inhibitor, RS102895 (40 μM) (Sigma-Aldrich) was added to the CM. RS102895 was dissolved by dimethylsulfoxide (DMSO). Cell migration across the wound surface was monitored by microscopy at various time points. Quantitation was performed by measuring the distance of the wound edge of migrating cells from the start point to the migration point. Experiments were performed in biological triplicate.

### Murine OIR model

Animal experiments were performed in accordance with the guidelines of the Japanese Society for Pharmacology and were approved by the Committee for the Ethical Use of Experimental Animals at Setsunan University, Osaka, Japan. OIR was induced in C57BL/6 mice as previously described [13]. In brief, at postnatal day (P) 7, pups and their nursing mothers were exposed to hyperoxic conditions (75 % oxygen) for 5 days. On P12, pups were returned to room-air (normoxic) conditions for 3 or 5 days until P15 or P17, respectively.

### In vivo delivery of siRNA

For in vivo RNAi experiments, 0.5 μl of Apelin or AllStar Negative siRNA (200 pmol) (Cat. No. 00900627 and 1027292, respectively; Qiagen, Valencia, CA) was combined with 0.5 μl of Invivofectamine Reagent (Invitrogen) and glucose (5 % final) and incubated for 30 min at room temperature. Mice were anesthetized via intraperitoneal injection with chloral hydrate (400 mg/kg), and a mixture of 0.5 % (v/v) tropicamide and 0.5 % (v/v) phenylephrine hydrochloride was applied to eye (Mydrin P; Santen Pharmaceutical Co. Ltd., Osaka, Japan). The RNAi mixture was injected intravitreally at postnatal day 12 and 15 (1 μl/eye).

### Immunohistochemistry and lectin staining

Retinas were fixed with 4 % paraformaldehyde/PBS solution and blocked in TBS containing 0.5 % Triton X-100 and 5 % FBS. Immunohistochemistry was performed using anti-NG2 chondroitin sulfate proteoglycan (Millipore Corp., Bedford, MA), anti-CCR2 (Abcam) or anti-integrin αM (M-19) (Santa Cruz Biotechnology) antibodies, and visualized with Alexa Fluor 568-conjugated goat anti-rabbit IgG (Molecular Probes) or Alexa Fluor 350-conjugated donkey anti-goat IgG (Molecular Probes). Lectin staining was performed using Alexa Fluor 488-conjugated *Griffonia (Bandeiraea) simplicifolia* isolectin B4 (IB4) (Molecular Probes). To evaluate the coverage of pericytes on newly formed vessels, 10 fields per retina were randomly selected at the leading edge of vessels. Macrophage density was quantified by collecting fluorescence images of retina.

### Quantification of vascular angiogenesis and neovascular tufts

Retinal angiogenesis was assessed as previously described [10]. In brief, mice were anesthetized and perfused with saline containing 40 mg/mL of fluorescein isothiocyanate-labeled dextran (molecular weight, 2,000,000, Sigma-Aldrich) through the left ventricle at P17. Subsequently, eyes were removed and fixed for 1 h in 4 % paraformaldehyde/PBS. Retinas were dissected and flat-mounted in Fluoromount (Diagnostic BioSystems). Photographs were taken with a fluorescence microscope (AZ-100 M, Nikon, Tokyo, Japan). The area of neovascular tufts was measured as described by Banin et al. [22]. In addition to FITC-dextran perfusion, IB4 staining was also performed to quantify the vascular area. For quantitation of nuclei extending beyond the internal limiting membrane, the eyes of mice were enucleated and fixed in 4 % paraformaldehyde/PBS for 24 h and embedded in paraffin. Six sections were selected within 300 μm of the optic nerve in serial sagittal sections (5 μm thickness) of whole eyes, and stained with hematoxylin–eosin (HE).

### Statistics

Data for Smad3 inhibitor on induction of MCP-1 expression by apelin siRNA, migration distance of VSMCs and mRNA expression studies in OIR model were analyzed using two-way ANOVA for treatments of siRNA or SIS3, treatments of conditional medium or CCR2 inhibitor or siRNA treatment and time (day) as the independent two factors, respectively, followed by the Tukey–Kramer test. The student’s *t* test for the others was used to assess statistical significance. A *p* value lower than 0.05 was considered statistically significant.

## Results

### Suppression of apelin expression leads to up-regulation of MCP-1 expression through activation of Smad3 via PI3K-Akt signaling in endothelial cells

**Fig. 1:**
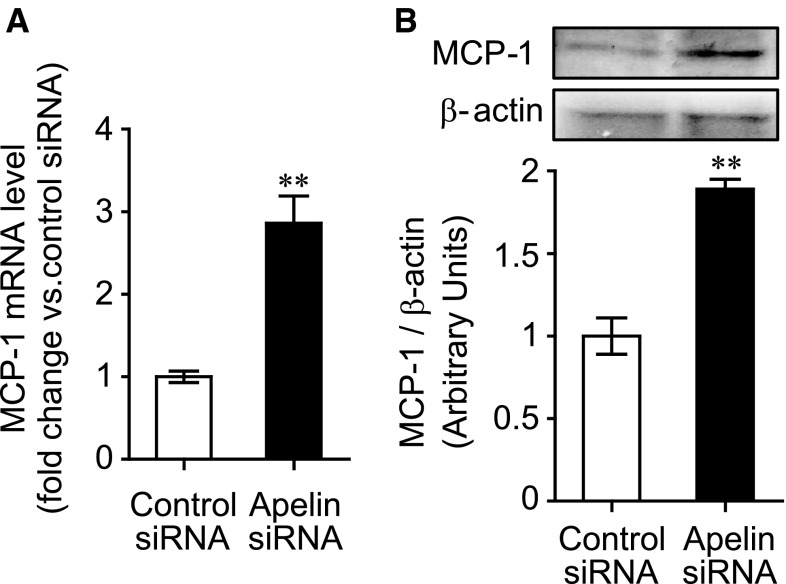
Apelin siRNA induces MCP-1 expression in endothelial cells. **a** Endothelial cells were exposed to siRNA transfection mixture for 24 h. MCP-1 mRNA expression was examined by real-time reverse-transcription polymerase chain reaction (RT-PCR) (n = 3). **b** MCP-1 protein expression was assessed in endothelial cells by western blot 48 h after siRNA transfection (n = 3). Data were analyzed by the student’s *t* test, and represent mean ± SEM. **p < 0.01 versus control siRNA

To investigate whether suppression of apelin expression accelerates pericyte recruitment, we first examined whether targeted knockdown of apelin using siRNA, influenced the expression of specific factors in bEnd.3 murine endothelial cells, which regulate vessel stabilization and pericyte recruitment [4, 19]. The expression of platelet-derived growth factor-B (PDGFB) and transforming growth factor β (TGF-β) in endothelial cells was not significantly affected following transfection with apelin siRNA after 24 h (PDGFB, 0.99 ± 0.04 fold change; TGF-β, 1.20 ± 0.13 fold change vs. control siRNA). In contrast, expression of MCP-1 was significantly up-regulated following treatment with apelin siRNA compared with control siRNA after 24 h (2.86 ± 0.33) (Fig. 1a). Furthermore, MCP-1 protein expression was also significantly up-regulated by apelin siRNA compared with control siRNA (1.89 ± 0.06 fold change vs. control siRNA) (Fig. 1b). We also examined the time course of apelin and MCP-1 expression after apelin siRNA treatment. The induction of MCP-1 expression was found at least 9 h later after apelin siRNA treatment (Supplemental Figure S1).

**Fig. 2:**
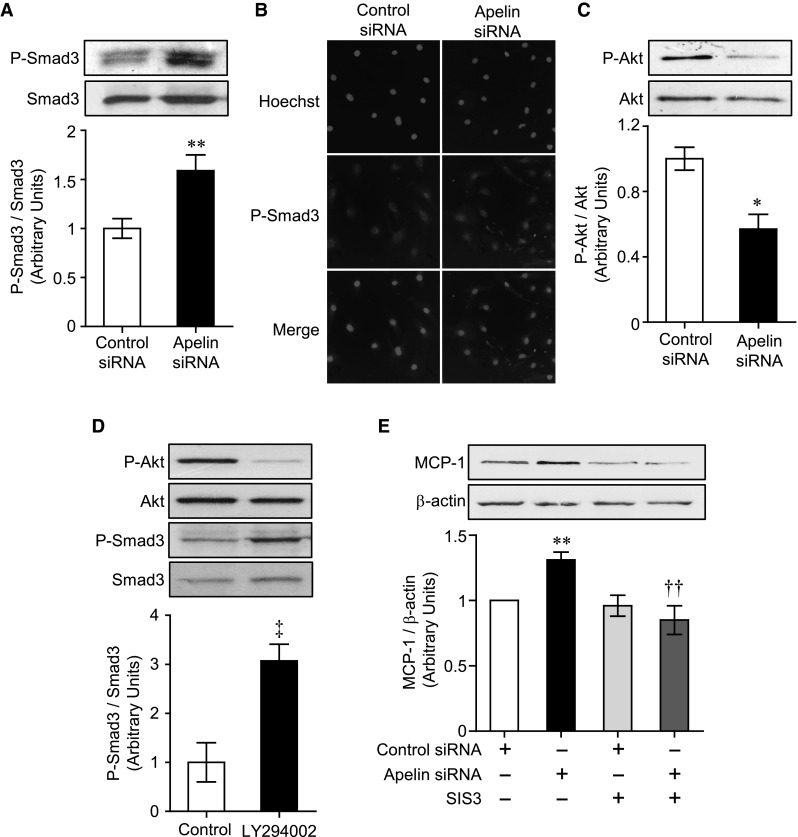
Suppression of apelin signaling leads to up-regulation of MCP-1 expression through activation of Smad3 via PI3K-Akt signaling in endothelial cells. **a** Levels of phosphorylated-Smad3 were assessed in endothelial cells by western blot 3 h after siRNA transfection (n = 3). **b** The translocation of phosphorylated-Smad3 in endothelial cells was examined by immunocytochemistry with anti-phosphorylated-Smad3 antibody (*red*). Nuclei were labeled with Hoechst 33342 (*blue*). **c** Effect of apelin siRNA on phosphorylated-Akt levels in endothelial cells was examined by western blot (n = 3). **d** Phosphorylated-Smad3 levels in endothelial cells treated with LY294002 (3 μM) for 3 h were detected by western blot (n = 3). **e** Effect of SIS3 on up-regulation of MCP-1 expression following treatment with apelin siRNA in endothelial cells was examined by western blot (n = 3). Cells were incubated with SIS3 (3 μM) for 1 h prior to transfection with siRNA. Data were analyzed by student’s *t* test (**a**, **c**, **d**) or two-way ANOVA followed by Tukey–Kramer test (**e**) and represent mean ± SEM. *p < 0.05 and **p < 0.01 versus control siRNA, ^‡^ *p* < 0.01 versus control, ^††^ *p* < 0.01 versus apelin siRNA. (Color figure online)

MCP-1 expression is regulated not only by nuclear factor kappa-light-chain-enhancer of activated B cells (NF-κB) [23], but also by the Smad family of transcription factors whose activities are induced by the TGF-β pathway [19]. Therefore, to further elucidate the mechanism of MCP-1 up-regulation by apelin siRNA, we examined the activation of NF-κB and Smad family members (Smad1/5, 2, and 3). Apelin siRNA did not affect nuclear translocation of NF-κB and phosphorylation of Smad1/5 and Smad2 (Supplemental Figure S2). In contrast, knockdown of apelin in endothelial cells induced Smad3 phosphorylation, while total levels of Smad3 remained unchanged (1.59 ± 0.16 fold change vs. control siRNA) (Fig. 2a). The phospho-Smad3 activated by apelin siRNA was subsequently translocated into the nucleus (Fig. 2b).

Apelin activates the PI3K-Akt pathway involved in cell proliferation [24, 25], and previous studies have shown that PI3K modulates Smad signaling [26]. Therefore, to investigate the involvement of these signaling pathways in the effect mediated by apelin knockdown, endothelial cells were treated with apelin siRNA or the PI3K inhibitor, LY294002. Apelin siRNA suppressed Akt phosphorylation, followed by induction of Smad3 phosphorylation (Fig. 2c). Treatment with LY294002 (3 μM) also suppressed Akt phosphorylation and induced Smad3 phosphorylation (Fig. 2d). In addition, treatment of endothelial cells with apelin siRNA for 24 h induced up-regulation of MCP-1 protein expression, and this was blocked by treatment with SIS3, a specific inhibitor of Smad3 (Fig. 2e). Furthermore, LY294002 treatment for at least 6 h significantly induced MCP-1 mRNA expression (1.65 ± 0.16 fold change vs. vehicle). These data suggest that the apelin-APJ system modulates Smad3 phosphorylation through PI3K-Akt signaling, followed by up-regulation of MCP-1 expression.

Since previous studies have shown that both antisense and siRNA agents may exert non-target-related biological effects including immune stimulation [27], we also examined if effect of the apelin blockade on expression of MCP-1 is off-target effects by siRNAs. Control siRNA did not change the phosphorylation of Akt or Smad3 compared with untreated control (Supplemental Figure S3).

### Conditioned medium from endothelial cells treated with apelin siRNA enhances the migratory activity of vascular smooth muscle cells

**Fig. 3:**
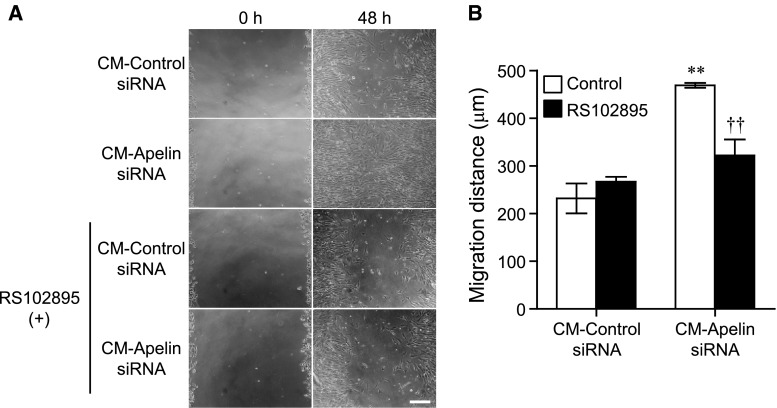
The conditioned medium (CM) of endothelial cells pre-treated with apelin siRNA enhances vascular smooth muscle cell migration. **a** Confluent VSMC monolayers were wounded by scraping and treated with CM from endothelial cells exposed to respective siRNAs for 48 h. The CM from siRNA-treated endothelial cells was added to VSMCs and cells were treated with RS102895 (40 μM) or DMSO. **b** Cell migration to the wound surface was monitored between 0 and 48 h. The distance of migration of the wound edge was quantitated (n = 3). CM-control siRNA and CM-apelin siRNA indicate CM from endothelial cells exposed to respective siRNAs. Bar represents 100 μm. Data were analyzed by two-way ANOVA followed by Tukey–Kramer test, and represent mean ± SEM. **p < 0.01 versus CM-control siRNA, and ^††^ *p* < 0.01 versus control

To determine whether induction of MCP-1 following knockdown of apelin plays a role in mural cell recruitment, we next investigated the migratory activity of VSMCs after exposure to CM prepared from apelin siRNA-treated endothelial cells. The CM from endothelial cells treated with apelin siRNA for 48 h significantly enhanced VSMC migration compared to that derived from control siRNA treated cells (Fig. 3). MCP-1 acts through the C–C chemokine receptor 2 (CCR2). Therefore, we next examined whether the MCP-1/CCR2 signal pathway mediates the enhancement of migration of VSMCs by using RS102895, a potent CCR2 antagonist. As shown in Fig. 3, RS102895 (40 μM) significantly blocked the promotion of VSMC migration by the CM from apelin siRNA-treated endothelial cells (Fig. 3). RS102895 did not influence the survival and migration of VMSCs and was not toxic at the concentration analyzed. These results suggest that CM from cells treated with apelin siRNA induces VSMC migration via the MCP-1/CCR2 signaling pathway.

### In vivo delivery of apelin siRNA promotes pericyte recruitment in the retinas of a murine model of oxygen-induced retinopathy

We next investigated whether knockdown of apelin also affected MCP-1 expression in vivo, using a murine model of OIR. To verify the distribution of siRNA in retinal tissue after intravitreal injection, we used Alexa-488 labeled apelin siRNA. Alexa-488 signal was detected in the surface of the retina including CD31-positive endothelial cells 48 h after intravitreal injection (Supplemental Figure S4, arrowheads). These results indicate that intravitreal injection with Invivofectamine can deliver siRNA into retinal tissues, and that siRNA is present in retinal tissues at least 2 days after transfection.

**Fig. 4:**
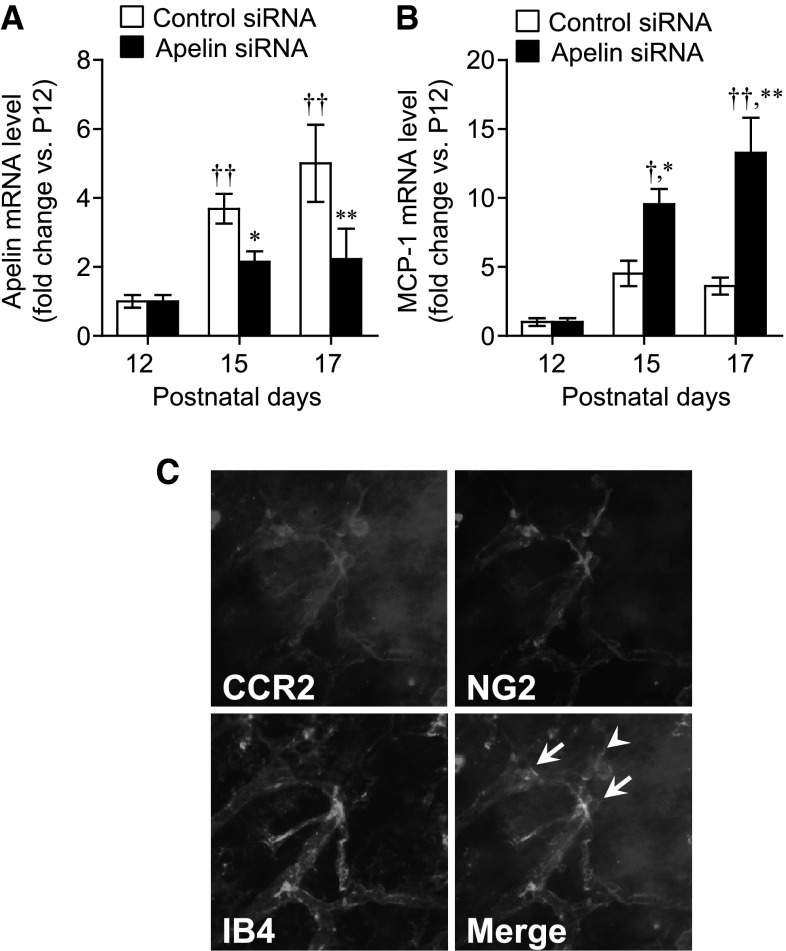
Expression of MCP-1 and its receptor CCR2, in the retinas of OIR model mice. The effect of apelin siRNA on the expression of apelin (**a**) and MCP-1 (**b**) in retinas of OIR model mice was examined by real-time RT-PCR (n = 7 to 9). **c** Representative pictures showing double immunostaining of CCR2 (*blue*) and NG-2 (*red*) combined with isolectin B4 (IB4) (*green*) staining in retina of OIR model mice at P17. *Arrows* indicate CCR2 and NG-2 double positive cells. Data were analyzed by two-way ANOVA followed by Tukey–Kramer test, and represent mean ± SEM. *p < 0.05 and **p < 0.01 versus control siRNA, ^†^ *p* < 0.05 and ^††^ *p* < 0.01 versus P12 value. (Color figure online)

Next, we evaluated the effect of apelin siRNA on MCP-1 expression in the retinas of mice with OIR. Intravitreal injection of apelin siRNA at P12 and P15 led to significant suppression of apelin mRNA at P15 and P17 compared with control siRNA (Fig. 4a). In keeping with the results of the in vitro assays, suppression of apelin mRNA led to a significant induction of MCP-1 in the retinas of mice with OIR, in a time-dependent manner (Fig. 4b). Moreover, we investigated whether these results was due to the off target response or the inflammatory response to siRNA injection in OIR model mice. While there was no difference in MCP-1 expression between control siRNA and uninjected control retina of OIR model, upregulation of MCP-1 expression was shown only by apelin siRNA in retinas of OIR model (Supplemental Figure S5). We also examined effect of apelin siRNA on microglial activation in avascular- and neovascular-area using an immunostaining of Iba-1, an activated microglia/macrophage marker. Although retinal macrophages in avascular and neovascular area of OIR at P17 seemed to be activated by even only transfection reagent, there was no difference in the activation of macrophages between control and apelin siRNA (Supplemental Figure S6). Since the role of MCP-1 in pericytes is largely unreported, we examined the expression of CCR2 in pericytes of retinas. Flat-mount immunostaining showed that CCR2 was localized with NG2-positive pericytes, but not IB4-positive endothelial cells (Fig. 4c, arrows).

**Fig. 5:**
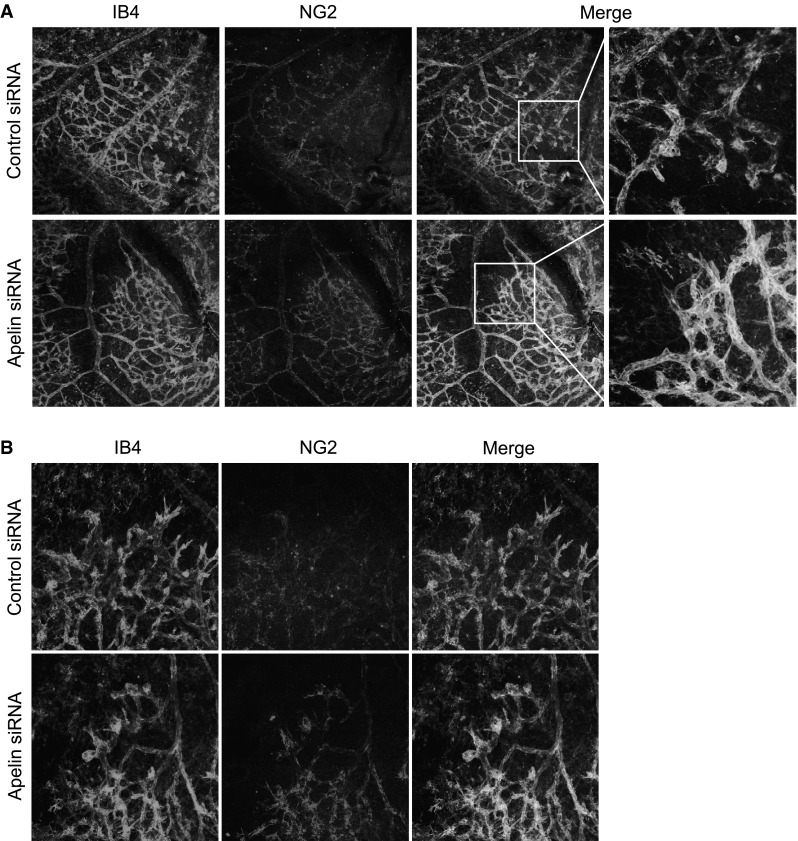
Apelin siRNA enhances pericyte coverage of newly formed vessels in OIR model mice. **a** Representative pictures show the leading edge of retinal vessels in OIR model mice treated with control siRNA (*upper panels*) or apelin siRNA (*lower panels*). **b** Representative images show pericyte coverage of the neovascular tufts in retinal vessels at P17 in OIR model treated with control siRNA (*upper panels*) or apelin siRNA (*lower panels*)

Next, we investigated whether apelin siRNA accelerated pericyte coverage on newly formed vessels in our OIR model. In comparison with control siRNA treated retinas, IB4 positive cells were more covered with NG-2 positive cells at the leading edge of retinal vessels at P17 in mice treated with apelin siRNA (Fig. 5a). When we quantified the coverage of NG-2 positive cells to retinal capillaries, the presence of NG-2 positive cells on newly formed vessels in retinas treated with apelin siRNA was significantly higher compared with treatment with control siRNA in this OIR model at P17 (control siRNA, 42.79 ± 1.05 %; apelin siRNA, 57.01 ± 2.65 %). In addition to the pericyte coverage at the leading edge of capillary, the pericyte coverage in the neovascular tuft of retinal vessels at P17 in mice treated with apelin siRNA was also higher than that with control siRNA (Fig. 5b). These results suggest that in vivo delivery of siRNA targeting apelin promotes pericyte recruitment in retinas of OIR mice.

### Apelin siRNA inhibits pathological retinal angiogenesis in OIR model mice

Abnormal vessel formation results from insufficient recruitment of mural cells, which contribute to the mechanical stability of the capillary wall [28]. Therefore, we evaluated the effect of apelin siRNA on pathological angiogenesis in retinas of OIR model mice using FITC-dextran perfusion. Intravitreal injection of apelin siRNA at P12 and P15 significantly reduced the area of the retinal capillary at P17 compared with contralaterally applied control siRNA (apelin siRNA, 32.98 ± 0.85 %; control siRNA, 40.02 ± 1.03 %) (Supplemental Figure S7A and B). In accordance with the capillary area, we observed a significant decrease in the expression of CD31 mRNA, an endothelial marker, in retinas treated with apelin siRNA (0.84 ± 0.05 fold change vs. control siRNA) (Supplemental Figure S7C). Moreover, IB4 staining showed that apelin siRNA significantly suppressed retinal angiogenesis in OIR model mice, while control siRNA did not change the capillary area compared with uninjected control retina (Supplemental Figure S8).

**Fig. 6:**
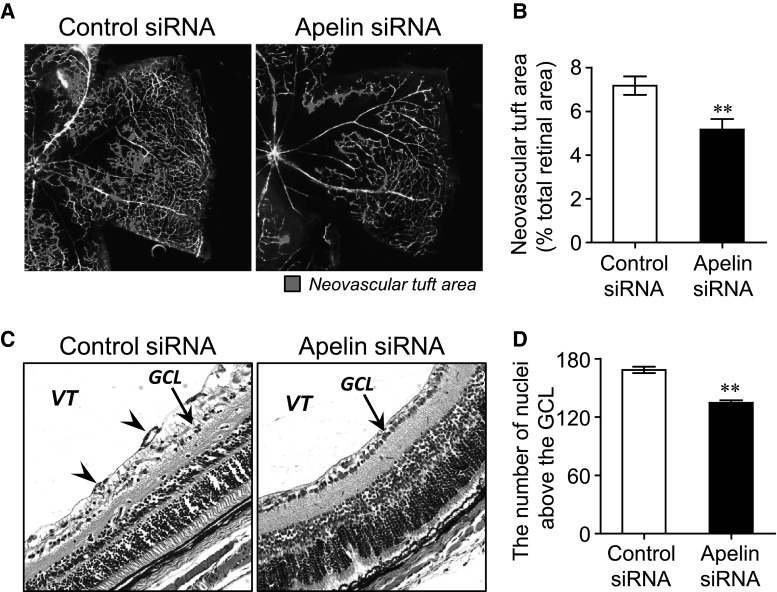
Apelin siRNA prevents pathological retinal angiogenesis in OIR model mice at P17. **a** Representative pictures of retinal flat-mounts from OIR model mice treated with apelin siRNA (*right panel*) or control siRNA (*left panel*). The neovascular tuft area is highlighted in *red*. **b** The percentage of neovascular tufts was calculated by dividing the neovascular tuft area by the retinal capillary area (n = 12). **c** Representative pictures showing HE staining of retinal cross-sections from OIR model mice treated with apelin siRNA (*right panel*) or control siRNA (*left panel*). **d** Intravitreal neovascularization was quantified by the number of cell nuclei growing beyond the GCL (n = 6). *VT* vitreous; *GCL* ganglion cell layer; arrowheads, vascular-like structures. Data were analyzed by student’s *t* test and represent mean ± SEM. **p < 0.01 versus control siRNA. (Color figure online)

To quantitatively assess pathological angiogenesis, we measured the total area of aneurysm-like structures >10 μm in retinal flat-mount and counted the number of vascular nuclei extending into the vitreous body from the retinal surface in retinal cross sections at P17. As shown in Fig. 6a, b, abnormal neovascular tuft formations at P17 were suppressed by treatment with apelin siRNA (apelin siRNA, 5.17 ± 0.49 %; control siRNA, 7.18 ± 0.42 %). HE staining of retinal cross sections from mice revealed that the number of vascular nuclei extending into the vitreous body from the retinal surface were also significantly reduced in apelin siRNA-injected eyes compared with contralaterally control siRNA (apelin siRNA, 134.72 ± 2.5; control siRNA, 168.50 ± 3.3) (Fig. 6c, d).

## Discussion

Here we provide the first evidence that the apelin-APJ system switches endothelial cells from proliferative to mature state in angiogenesis. We demonstrate that apelin knockdown induces Smad3 phosphorylation and nuclear translocation via suppression of PI3K/Akt signaling, followed by up-regulation of MCP-1 expression, thus promoting pericyte recruitment. We also show that in vivo delivery of apelin siRNA results in increased pericyte coverage of newly formed vessels and suppresses pathological angiogenesis. Therefore, we concluded that apelin is not only a key regulator of endothelial cell growth but also the initial trigger for pericyte recruitment during pathological retinal angiogenesis.

**Fig. 7:**
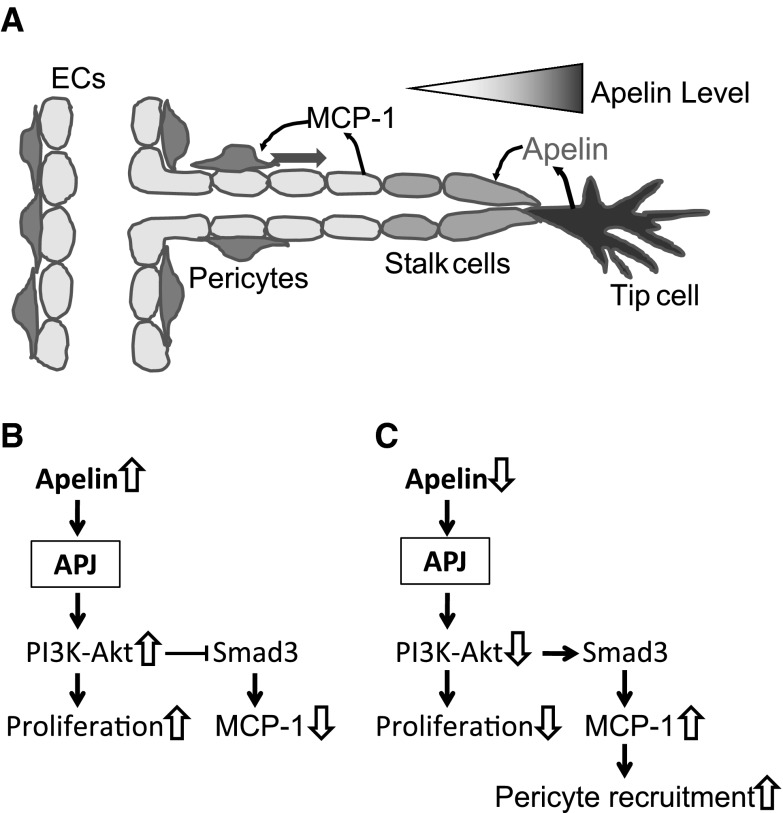
Proposed mechanisms for the apelin-APJ system on endothelial cells during angiogenesis. **a** Endothelial cells moving away from the tip cells receive apelin signals since apelin is selectively secreted from the tip cells. **b** The apelin-APJ system stimulates proliferation of the stalk cells through PI3-Akt signaling, and inhibits MCP-1 expression by suppression of Smad3 phosphorylation. **c** Reduction of this signaling attenuates proliferation of endothelial cells at a site distant from the tip cells, and releases the inhibition of Smad3 cascade, followed by the release of MCP-1 for maturation of vessels

It is widely accepted that maturation of blood vessels is closely related to cell contact between mural cells and endothelial cells. However, to date, it is not clear when the factors of pericyte recruitment are released after the formation of the endothelial lumen. In addition to the excessive expression of apelin in the OIR model, apelin secreted from the tip cells binds to APJ on endothelial stalk cells and induces their proliferation [29]. Reduction of growth signaling due to the physical separation may constitute the primary mechanism of the next step. Therefore, we hypothesized that endothelial cells moving away from tip cells rarely receive proliferative signals from the apelin-APJ system, and that the reduction of proliferative signaling initiates the maturation process (Fig. 7a). In keeping with this model, suppression of apelin signaling by siRNA both in vivo and in vitro significantly induced MCP-1 expression which recruits mural cells to endothelial cells [19, 20]. Moreover, the reduction in neovascular tuft-associated macrophages/microglia in MCP-1 deficient mice correlates with vascular remodeling (tuft apoptosis and regression of retinal neovascularization), not with neovascular response, in the OIR model [30]. Taken together, we propose a mechanism for the interaction between the apelin-APJ system and pericyte recruitment. While the apelin-APJ system promotes endothelial proliferation and suppresses MCP-1 expression in stalk cells, which receive a strong signal from the apelin-APJ system (Fig. 7b), reduction of this signaling results in the attenuation of cell proliferation and the release of pericyte recruitment factor in endothelial cells at a site distant from tip cells (Fig. 7c).

The question arises regarding why treatment with apelin siRNA specifically induced MCP-1 expression among the recruitment factors for pericytes in this study. In many studies including ours, endothelial cells were cultured under high glucose conditions (4.5 g/L). Expression of MCP-1 in the retina of ice with OIR was also up-regulated following injection of apelin siRNA in conjunction with 5 % glucose (Fig. 4c). High glucose increases endothelial TGF-β secretion [31]. Smad3 is one of several intracellular mediators capable of transducing signals from TGF-β receptors, and regulates transcription of target genes [32]. Therefore, to demonstrate that the effect of apelin siRNA treatment is mediated via the TGF-β pathway, we examined whether the effect of apelin siRNA on MCP-1 expression was diminished under low glucose conditions. As expected, upregulation of MCP-1 expression induced by apelin siRNA was diminished under low glucose conditions (Supplemental Figure S9). In addition to the results showing that apelin siRNA leads to a reduction in Akt phosphorylation, treatment with Smad3 inhibitor blocked MCP-1 up-regulation by apelin siRNA. These data indicate that the apelin-APJ system mediates Smad3 activation through the PI3K-Akt pathway, followed by regulation of MCP-1 expression.

Intriguingly, a recent study showed that stimulation of endothelial cells by either bone morphogenetic protein (BMP)-9 or -10, led to a decrease in apelin expression [33]. Moreover, treatment with apelin enhanced cardiac differentiation of murine and human embryonic stem cells and exhibited synergistic effects with mesodermal differentiation factors, such as BMP-4 [34]. Retardation of retinal vascular development is observed in both apelin-deficient and BMP-9/10 signaling-blocked mice [33]. Thus, these data imply a possible cross-talk between the apelin-APJ and BMP pathways, a main member of the TGF-β superfamily [35]. In this study, inhibition of the apelin-APJ signaling pathway induced Smad3 phosphorylation for TGF-β signaling cascade. Since the intracellular antagonism between BMP and TGF-β signaling might be mediated through sequestration of a limited pool of shared Smad protein [36], inhibition of the apelin-APJ system would inhibit BMP signaling through regulating TGF-β signaling. Taken together, our study demonstrating that the apelin-APJ system modulates Smad3 phosphorylation through PI3K-Akt signaling provides the evidence of cross-talk between the apelin-APJ and BMP, a TGF-β superfamily, signaling pathways.

We previously reported that the apelin-APJ system is a potent endothelial growth signal during pathological retinal angiogenesis using apelin deficient mice [13]. Pathological angiogenesis in growing tumors is induced by sustained Akt signaling [37]. The apelin-APJ system induces the Akt/p70S6 kinase pathway in endothelial cells [25]. Retinal apelin was dramatically increased in the OIR model, and the increase in apelin expression was higher than that of VEGF in the retinas of these mice [13]. In this manuscript, using a murine model of OIR, we also demonstrate that suppression of apelin expression significantly inhibits pathological angiogenesis without reduction of both VEGF mRNA and its protein expression (Supplemental Figure S10). These data strongly suggest that excessive expression of apelin causes exuberant endothelial cell growth and pathological retinal angiogenesis.

Abnormal vessel formation results from inappropriate levels of angiogenic molecules, whose spatio-temporal patterns of expression and concentration are tightly regulated [38], and insufficient recruitment of mural cells (smooth muscle cells and pericytes), which contribute to the mechanical stability of the capillary wall [28]. In addition, an insufficient number of microvascular pericytes leads to endothelial hyperplasia and abnormal vascular morphogenesis [28, 39]. Moreover, lack of MCP-1, a pericyte recruitment factor, delays regression of vascular tufts in the OIR model, implying that pericyte recruitment is dependent on MCP-1 signaling in this model [30]. In addition, abrupt endothelial proliferative activity increases vascular fragility and the propensity to hemorrhage, and causes detachment of pericytes [40]. Therefore, we conclude that not only the suppression of endothelial cell proliferation but also the promotion of pericyte recruitment at the leading edges of endothelial sprouts may be contributed to the reduction of abnormal neovascular tufts following apelin siRNA treatment in the OIR model.

Regarding the role of MCP-1, the effects on macrophages cannot be ignored since macrophages profoundly affect phenotypes in the OIR model [41, 42]. Therefore, we assessed the effect of apelin knockdown on macrophage accumulation in the retinas of OIR mice. The population of macrophages in the retina was slightly, but not significantly, increased by apelin siRNA treatment (control siRNA, 8.41 ± 0.54 %; apelin siRNA, 10.32 ± 0.77 %). The most likely explanation is that up-regulation of MCP-1 by apelin siRNA would be occurred at the local area because apelin and APJ expressions were restricted in the tip cells or the endothelial cells in newly formed vessels. Therefore, one explanation for the lack of increased macrophage influx in the retinas treated with apelin siRNA may be that the local up-regulation of MCP-1 did not mainly cause of macrophage accumulation from periphery.

Endothelial cell aggregation may be an important mechanism for apelin-mediated enlargement or maturation of blood vessels in peripheral pathological angiogenesis, including the tumor vasculature [11, 43]. On the other hand, endothelial cell aggregation due to excessive proliferation results in neovascular tufts in the retina [13]. As described above, the same factor may give rise to different events in the retina and peripheral tissues. Kidoya et al. [11] showed that apelin suppresses VEGF-induced vascular edema, although apelin alone did not affect vascular endothelial (VE)-cadherin-mediated cell–cell junctions. In the present study, treatment of endothelial cells with apelin siRNA did not induce VE-cadherin in accordance with this previous study (supplemental Figure S11). The different roles of the apelin-APJ system in retina and peripheral tissues could be due to components, such as astrocytes, related to angiogenesis [44, 45].

Pathological angiogenesis in patients with ischemic retinal diseases such as diabetic retinopathy results in visual loss and blindness, because the newly formed vessels are leaky and may cause vitreous hemorrhage [46]. Abnormal vessels, such as microaneurysms, are induced in patients with diabetic retinopathy [47]. These findings suggest that increasing pericyte coverage of capillaries in addition to inhibiting angiogenesis is important for the treatment of ischemic retinal diseases. Our data demonstrate that suppression of apelin expression has pleiotropic effects for endothelial cells and pericytes. Thus, therapeutic targeting of the apelin-APJ system may represent a novel approach for ischemic retinopathy associated with leaky vessels.

In conclusion, this is the first experimental study to demonstrate that inhibition of the apelin-APJ system facilitates retinal vessel maturation in an in vivo ischemic retinopathy model. Mechanistically, this occurs by promoting pericyte recruitment through induction and secretion of MCP-1 via Smad3 activation in endothelial cells, and is effective for suppressing abrupt vessel growth. These data establish a new connection between endothelial cell proliferation and mural recruitment under pathological conditions.

## Electronic supplementary material

Below is the link to the electronic supplementary material.

Supplementary material 1 (PDF 3428 kb)
