# Supplementary material for: Inhibition of apelin expression switches endothelial cells from proliferative to mature state in pathological retinal angiogenesis
Source: Angiogenesis. 2013 May 3;16(3):723–34. doi: 10.1007/s10456-013-9349-6 (PMC3682100; doi:10.1007/s10456-013-9349-6)
Supplement: Supplementary file 1 — Supplementary material 1 (PDF 3428 kb) [file 10456_2013_9349_MOESM1_ESM.pdf]

Table S1. The primer sets used for real-time RT-PCR analysis.

Gene	Forward	Reverse
Apelin	5'-GTT GCA GCA TGA ATC TGA GG-3'	5'-CTG CTT TAG AAA GGC ATG GG-3'
VEGF	5'-GGA GAC TCT TCG AGG AGC ACT T-3'	5'-GGC GAT TTA GCA GCA GAT ATA AGA A-3'
PDGFB	5'-GAA TCA GGC ATC GAG AGA GAC G-3'	5'-GCA AGA CTG TGG GCA GGG TTA T-3'
TGF-β	5'-CAG TGG CTG AAC CAA GGA GAC-3'	5'- ATC CCG TTG ATT TCC ACG TG-3'
MCP-1	5'-CCA CTC ACC TGC TGC TAC TCA T-3'	5'-TGG TGA TCC TCT TGT AGC TCT CC-3'
CD31	5'-AGA GAC GGT CTT GTC GCA GT-3'	5 ' -TAC TGG GCT TCG AGA GCA GT-3'
VE-cadherin	5'-TCC TCT GCA TCC TCA CTA TCA CA-3'	5'-GTA AGT GAC CAA CTG CTC GTG AAT-3'
ACTB	5'-AGT GTG ACG TTG ACA TCC GTA-3'	5'-GCC AGA GCA GTA ATC TCC TTC T-3'

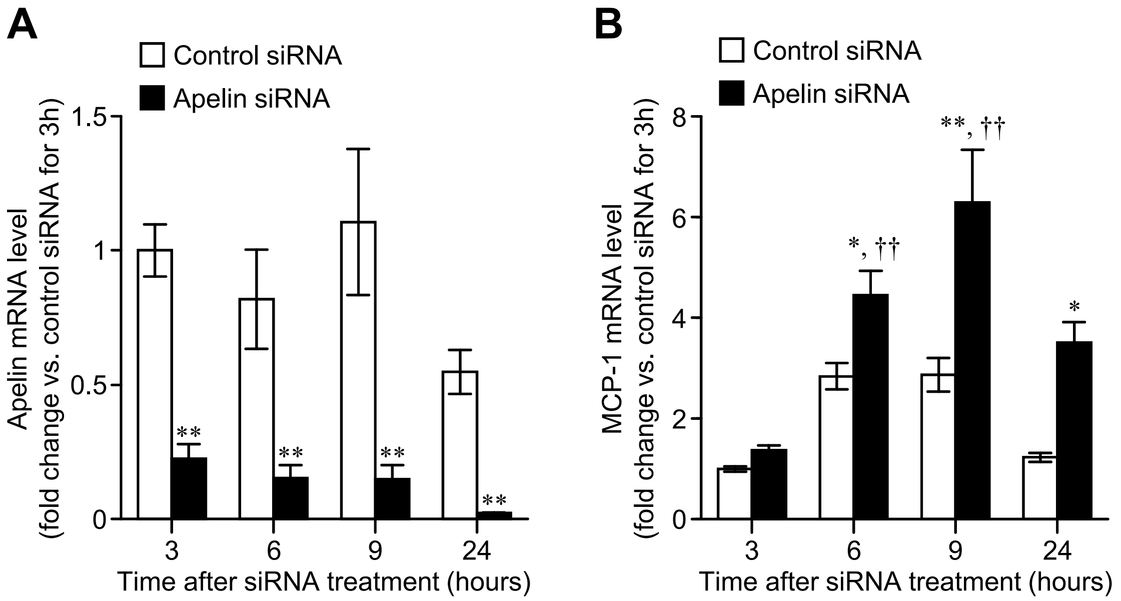

Supplemental Figure S1. Effect of apelin siRNA on gene expression in endothelial cells. Temporal gene expression of apelin (A) and MCP-1 (B) in endothelial cells exposed to apelin siRNA or control siRNA were examined by real-time RT-PCR method (n = 3 to 4). Data represent mean ± SEM. $^{*}p < 0.05$ and $^{**}p < 0.01$ vs. control siRNA; and $^{\dagger\dagger}p < 0.01$ vs. 3 hours.

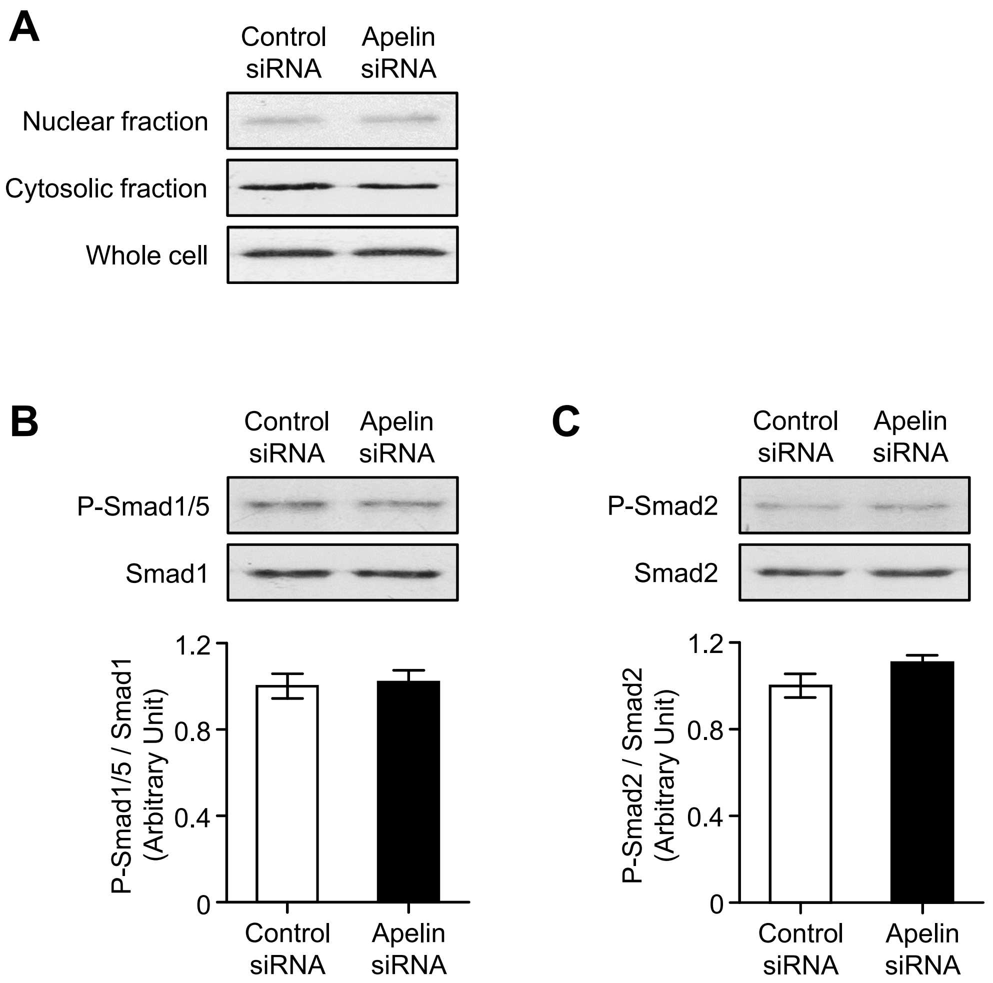

Supplemental Figure S2. Apelin siRNA exerts no influence on the translocation of NF-κB (A), and the activation of Smad1/5 (B) and Smad2 (C) in endothelial cells (n = 3). Primary antibodies were used anti-NF-κB p65 (C-20) (Santa Cruz Biotechnology), phospho-Smad1/5 (Ser463/465) (41D10) Rabbit mAb (Cell Signaling), Smad1 Antibody (Cell Signaling), phospho-Smad2 (Ser465/467)(138D4) Rabbit mAb (Cell Signaling) or Smad2 (D43B4) XP™ Rabbit mAb (Cell Signaling). Data represent mean ± SEM.

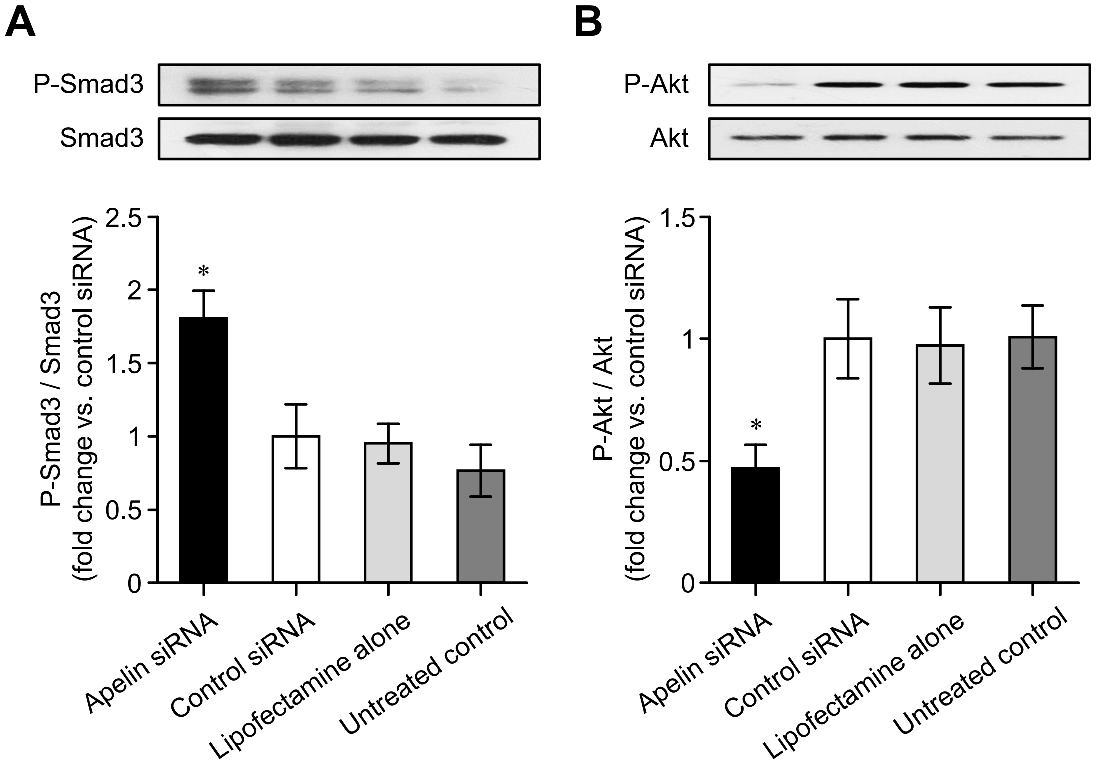

Supplemental Figure S3. The effect of apelin siRNA on the phosphorylation of smad3 (A) or Akt (B) protein. Control siRNA exerts no influence on the phosphorylation of Smad3 and Akt in endothelial cells compared with untreated control (n = 3). Data represent mean ± SEM. $^{*}p < 0.05$ vs. control siRNA

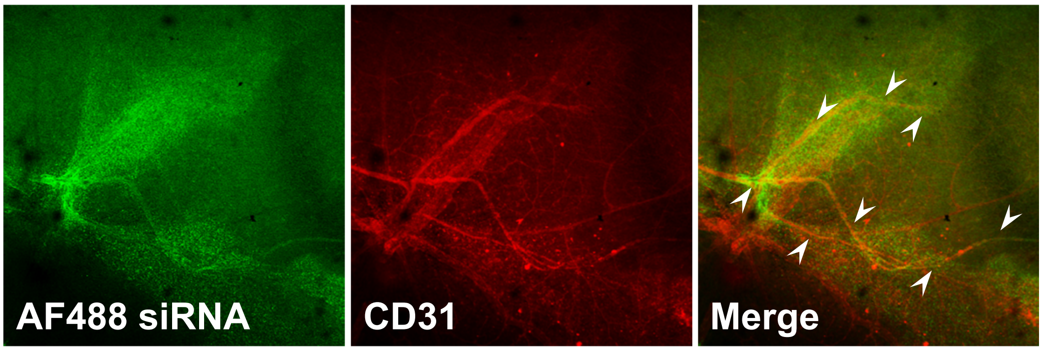

Supplemental Figure S4. *In vivo* delivery of siRNA to the retinas by intravitreal injection. Representative pictures of retinal central area show that strong green fluorescence was still observed in retinal surface 2 days after intravitreal injection of Alexa fluor 488 conjugated siRNA (AF488 siRNA). The Alexa fluor 488 signal was detected in the retinal surface including CD31 immunoreactive cells (arrowheads). CD31 antigen were probed with Purified Rat Anti-Mouse CD31 (BD Biosciences), and visualized with Polyclonal Rabbit Anti-Rat IgG/Biotinylated (Dako Cytomation) combined with streptavidin–Alexa Fluor 568 (Molecular probes).

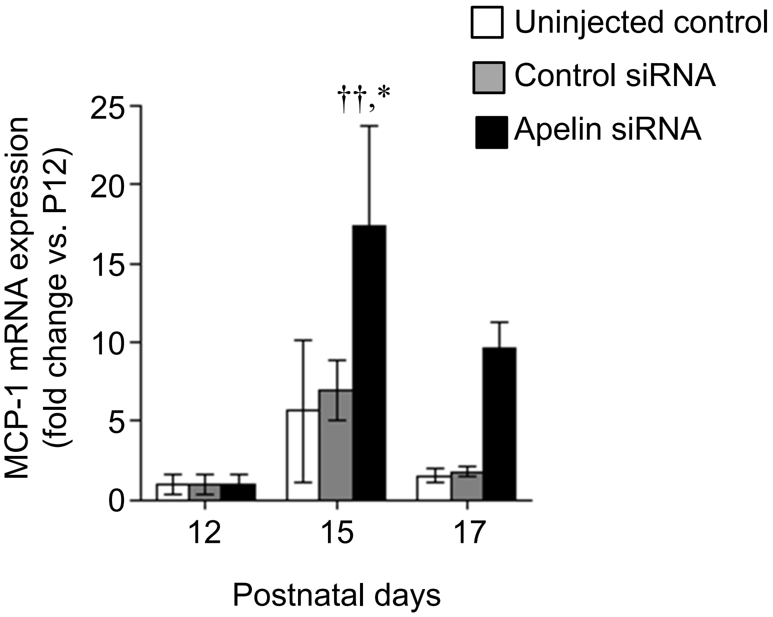

Supplemental Figure S5. Upregulation of MCP-1 expression by apelin siRNA is not due to off-target effects of siRNA in retinas of OIR model. MCP-1 expression was increased by apelin siRNA, not by control siRNA, compared with siRNA-*un*injected control retinas of OIR model (n = 4 to 7). Data represent mean ± SEM. *$p < 0.05$ vs. untreated control. ††$p < 0.01$ vs. P12.

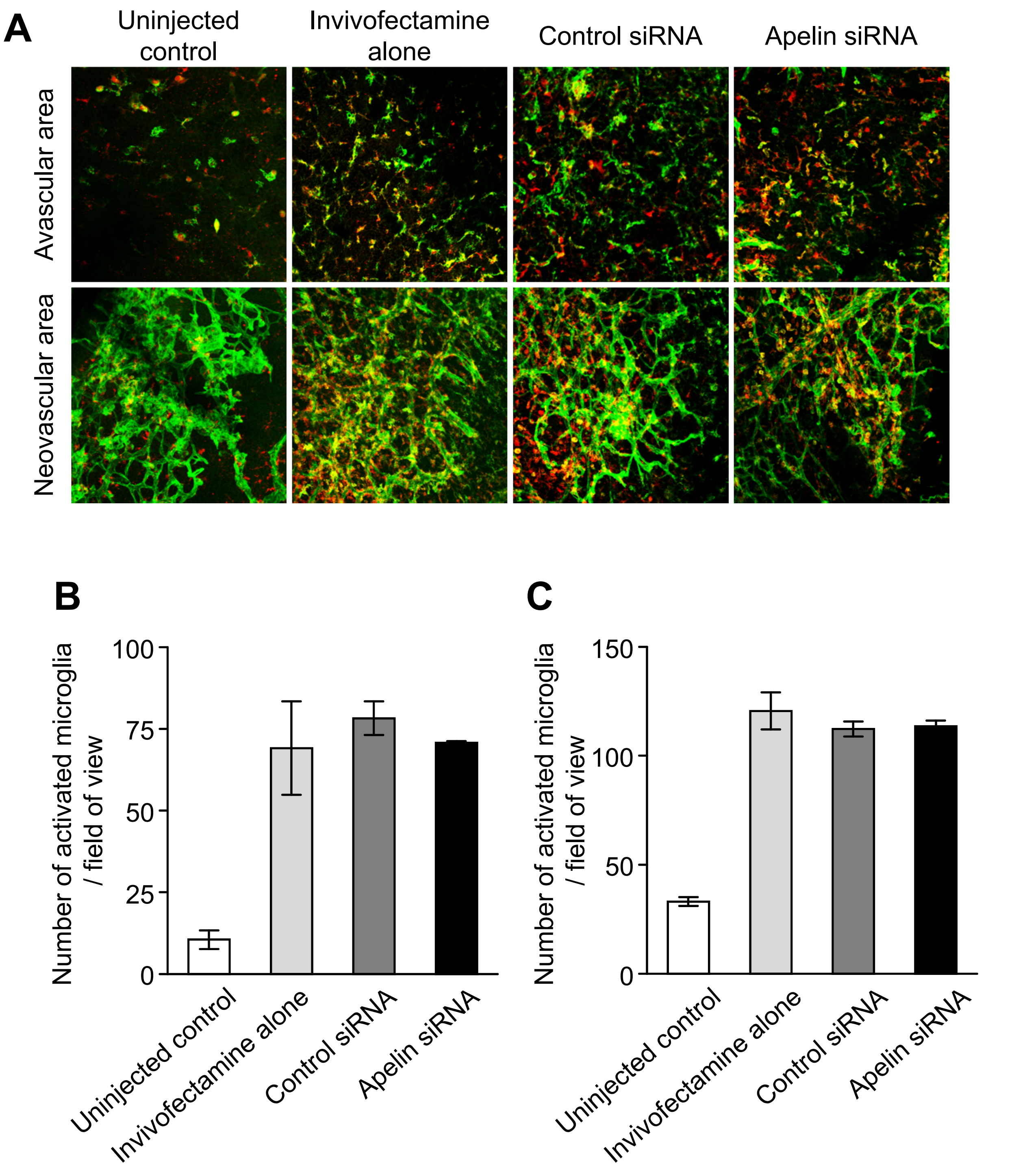

Supplemental Figure S6. Apelin siRNA exert no influence on the microglial activation induced by intravitreal injection of transfection reagent. (A) Representative pictures show double immunostaining of Iba-1 (red) and isolectin B4 (IB4) (green) in the avascular area (upper panels) and the neovascular area (lower panels) in retinas of OIR mice at P17. Iba-1 and IB4 double positive cells indicate activated microglia. Number of activated microglia was counted in the avascular area (B) and the neovascular area (C) (each treatment; n = 3).

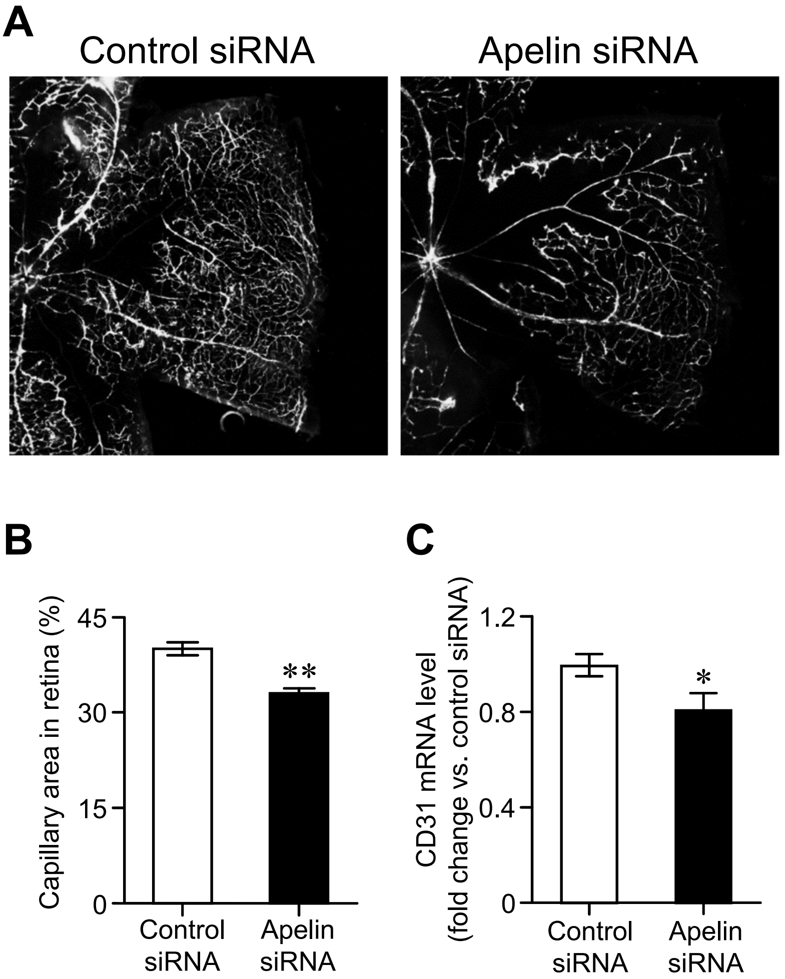

Supplemental Figure S7. Apelin siRNA suppresses retinal angiogenesis in OIR model mice at P17. (A) Representative pictures show retina treated with apelin siRNA (right panel) or control siRNA (left panel). (B) Capillary density in FITC-dextran perfused retinas of OIR model mice was quantified (n = 4). (C) Expression of CD31 mRNA in retinas of OIR model mice at P17 were (n = 5). Data represent mean ± SEM. *p < 0.05 and **p < 0.01 vs. control siRNA.

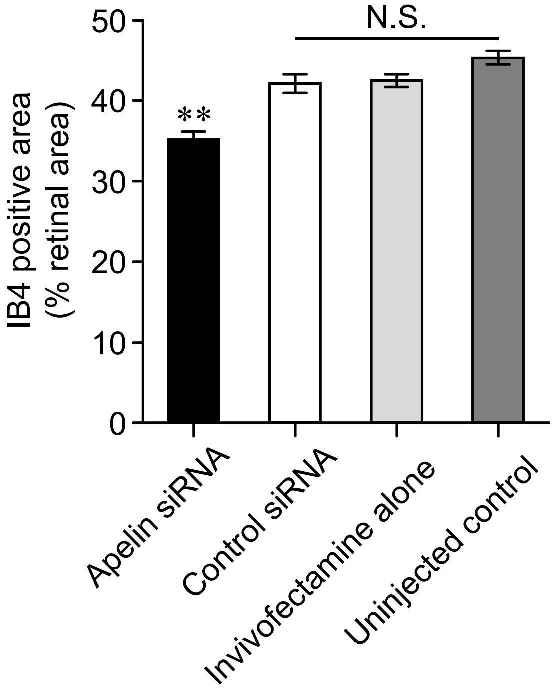

Supplemental Figure S8. Suppression of retinal angiogenesis by apelin siRNA is not due to off-target effects of siRNA. IB4 staining of whole mount retina at P17 shows that retinal angiogenesis in OIR model mice was suppressed by apelin siRNA, not by control siRNA and transfection reagent (n = 8 to 12). Data represent mean ± SEM. $**p < 0.01$ vs. control siRNA. N.S., no significant difference.

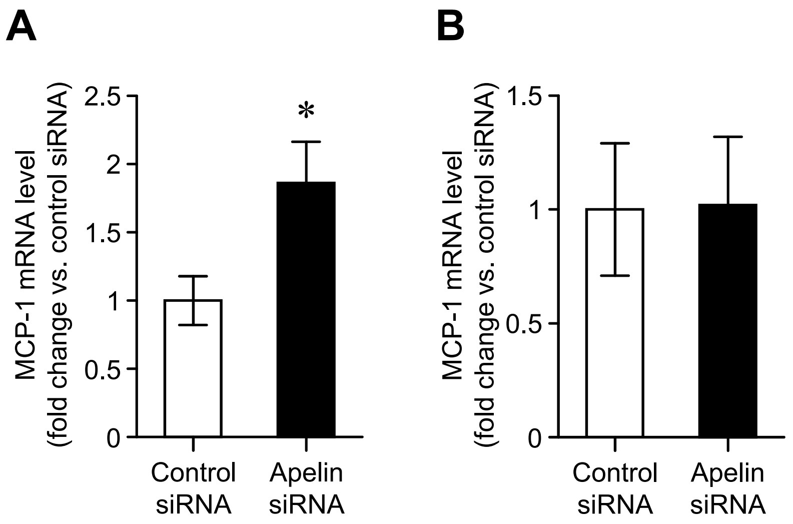

Supplemental Figure S9. Induction of MCP-1 expression by apelin siRNA in endothelial cells depends on the glucose concentration. Endothelial cells were cultured in high glucose medium (4.5 g/L) (A) or low glucose medium (1 g/L) (B). MCP-1 mRNA expression in endothelial cells was examined by real-time RT-PCR analysis (n = 5). Data represent mean ± SEM. **p* < 0.05 vs. control siRNA.

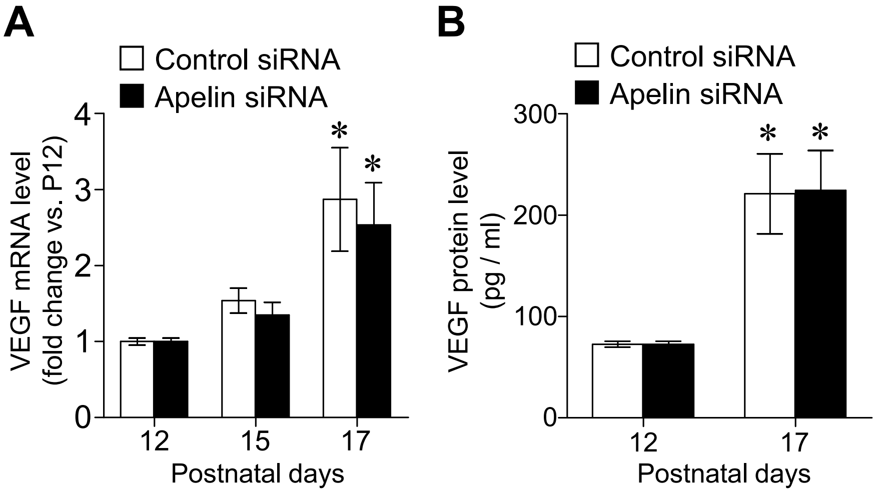

Supplemental Figure S10. Effect of apelin siRNA on VEGF expression in retinas of OIR model mice. (A) VEGF mRNA expression was quantified by real-time RT-PCR analysis (n = 7 to 9). (B) VEGF protein expression was assessed by ELISA (n = 5 to 6). Data represent mean ± SEM. *p < 0.05 vs. P12 value.

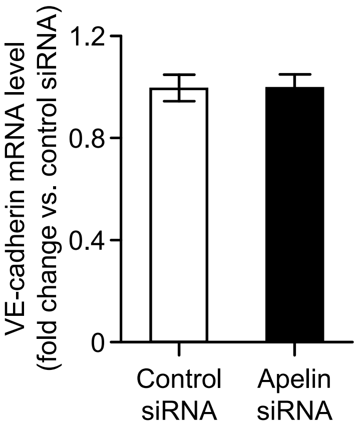

Supplemental Figure S11. Effect of apelin siRNA on VE-cadherin expression in retinas of OIR model mice at P17. VE-cadherin mRNA expression of retina was examined by real-time RT-PCR analysis (n = 5). Data represent mean ± SEM.
